# Supplementary material for: Monitoring peripheral nerve degeneration in ALS by label-free stimulated Raman scattering imaging
Source: Nat Commun. 2016 Oct 31;7:13283. doi: 10.1038/ncomms13283 (PMC5095598; doi:10.1038/ncomms13283)
Supplement: Supplementary Information — Supplementary Figures 1-20 and Supplementary Table 1. [file ncomms13283-s1.pdf]

Supplementary Information

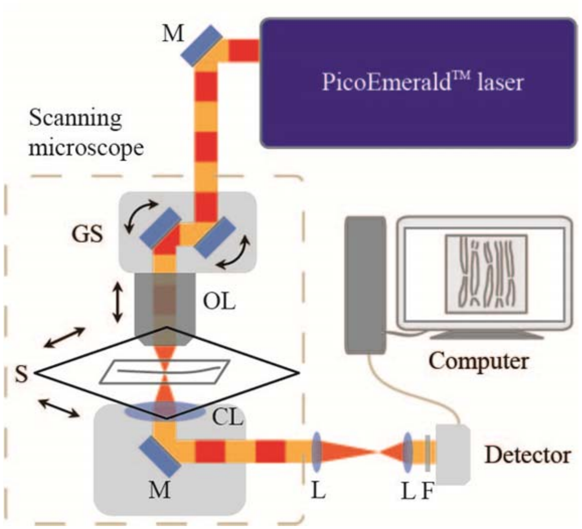

Supplementary Figure 1 Optical alignment for *ex vivo* SRS imaging of sciatic nerves. M: mirror, GS: galvo scanners, OL: objective lens, S: motorized translation sample stage, CL: condenser lens, L: lens, F: filter. Spatial and temporal overlapped lasers were sent to Olympus FV300 scanning microscope. The lasers were scanned by x-y galvo scanners and were focused on the sample by high numerical aperture (NA) water immersion objective. The z-direction movement was achieved by the z-direction movement of objective. The transmitted light through the sample was collected by a high NA condenser and went through bandpass filter (CARS 890/220). The pump laser was then measured by amplified photodiode (**Supplementary Fig. 20**) to generate electronic signal. The electronic signal was then processed by a lock-in amplifier and the output signal from lock-in amplifier was visualized on a computer monitor.

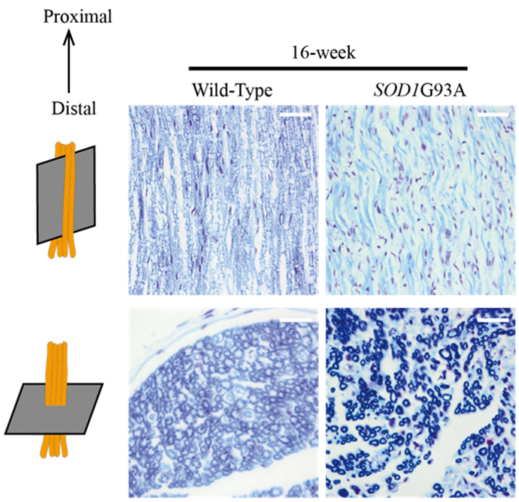

Supplementary Figure 2 Histology images of sciatic nerve of end-stage *SOD1*G93A and age-matched non-transgenic mice by Toluidine Blue staining. Scale bar, 50 µm.

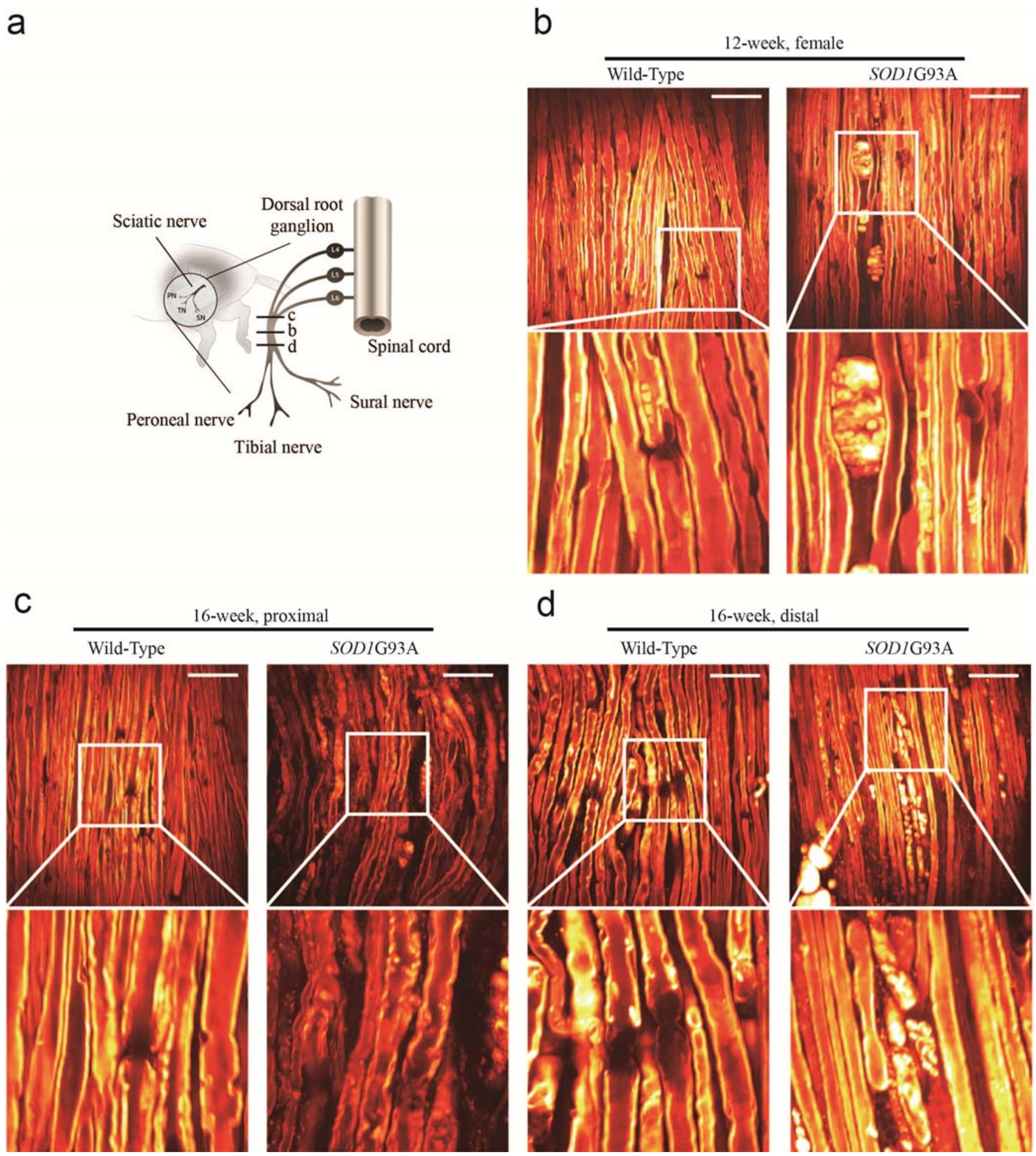

Supplementary Figure 3 Regional and sexual comparison of 16-week *SOD1*G93A mouse ALS model. (a) Experimental design. (b) Female, 12-week. Scale bar, 50 µm. (c) Proximal. Scale bar, 50 µm. (d) Distal. Scale bar, 50 µm.

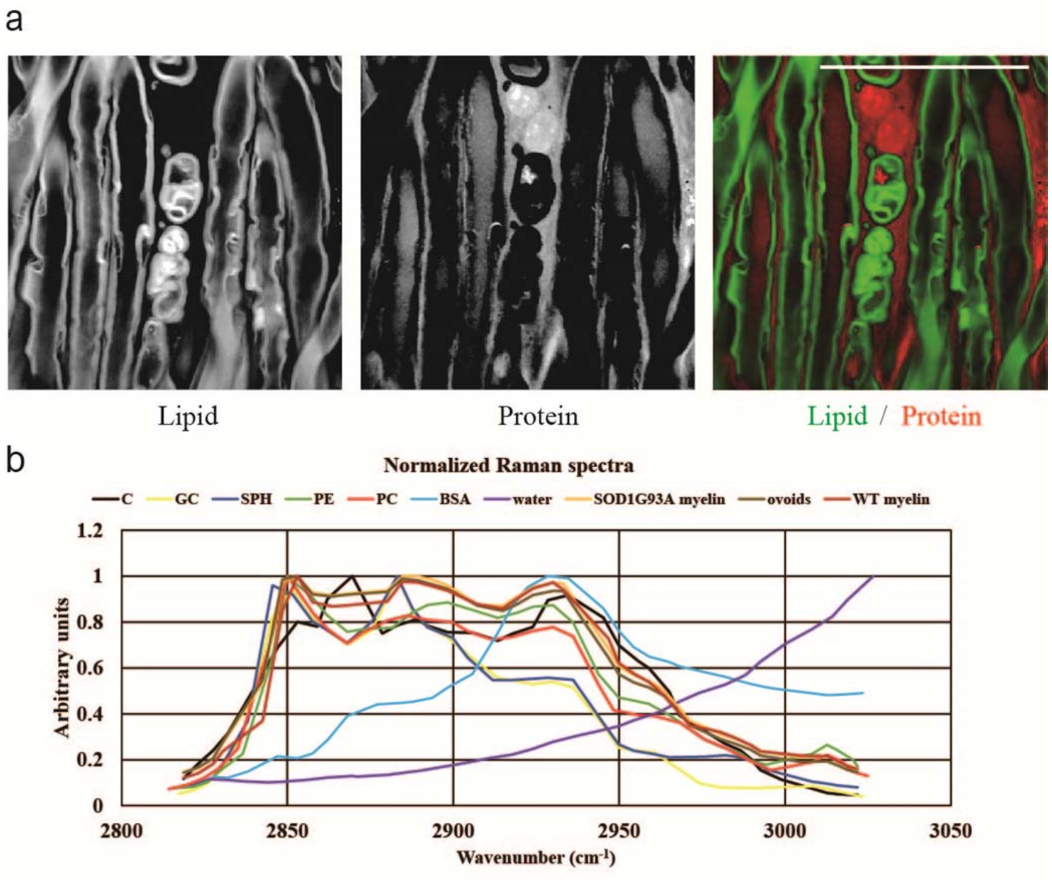

Supplementary Figure 4 Two color SRS imaging of sciatic nerve of *SOD1*G93A mouse and SRS spectrum of lipid and protein. (a) Two-color image of a typical lipid ovoid (from an 8-week *SOD1*G93A mouse). The protein image is the subtraction of image at 2850 cm^{-1} and 2950 cm^{-1}, after normalization with respect to pump power. Scale bar, 50 µm. (b) Spectra of standard chemicals and WT myelin, *SOD1G93A* myelin and *SOD1G93A* lipid ovoids. Analyzed chemicals have been abbreviated as cholesterol (C), galactocerebroside (GC), sphingomyelin (SPH), phosphatidylcholine (PC), phosphatidylethanolamine (PE) and bovine serum albumin (BSA).

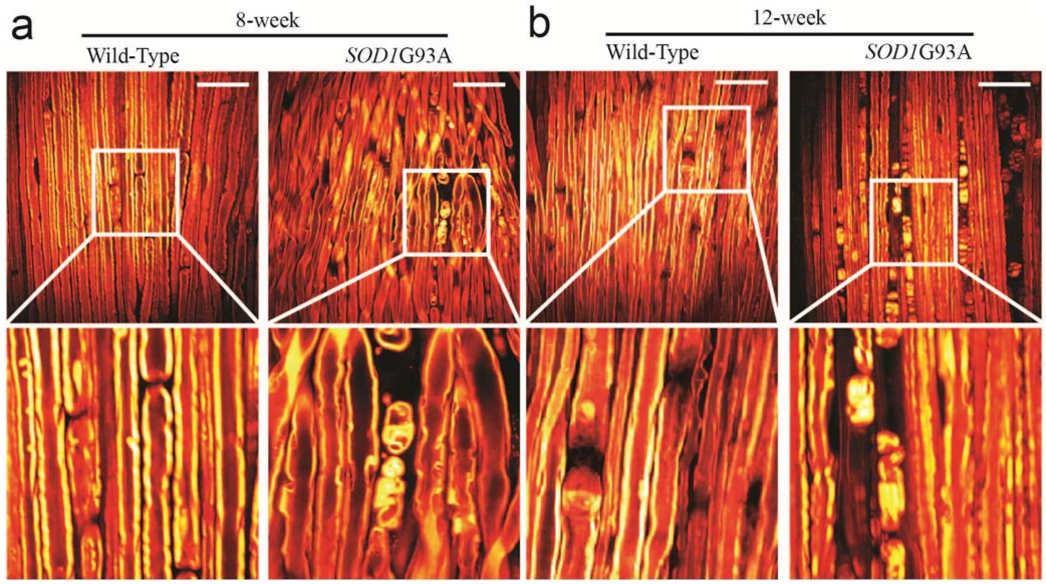

Supplementary Figure 5 *Ex vivo* sciatic nerve SRS images from *SOD1*G93A transgenic versus WT non-transgenic mice at the age of 8 weeks (a) and 12 weeks (b). Scale bar, 50 µm.

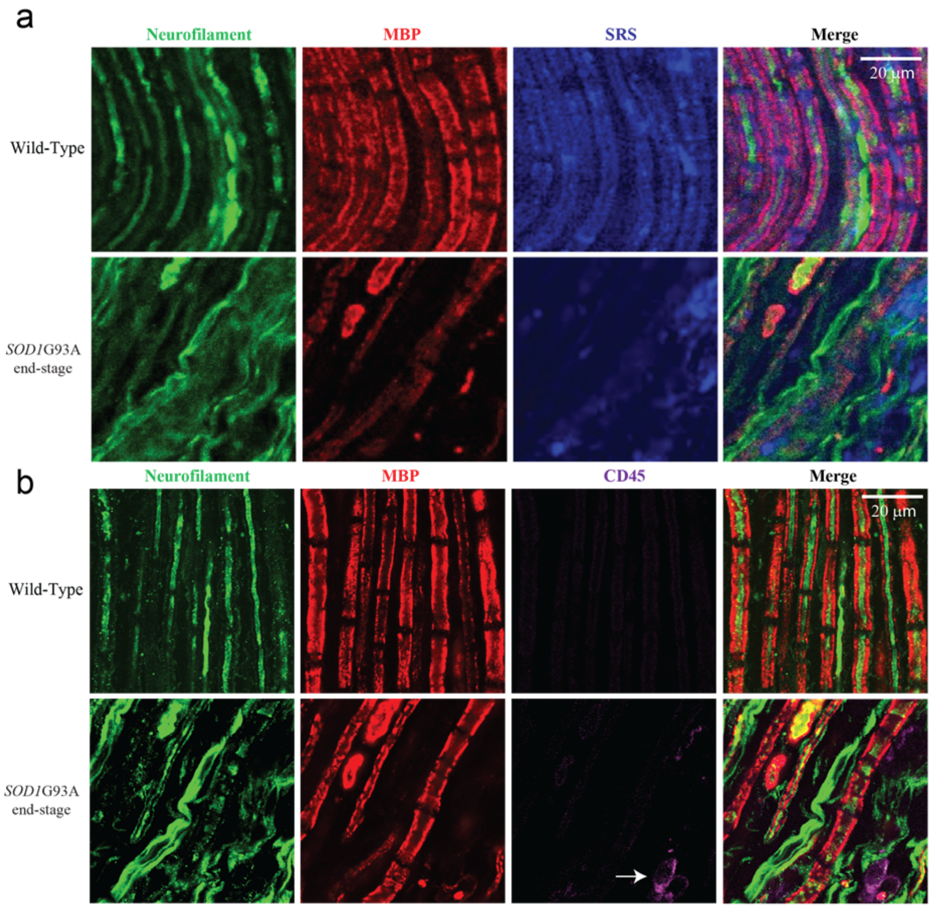

Supplementary Figure 6 Whole mount immunostaining of sciatic nerves from 16-week *SOD1*G93A versus WT. (a) SRS lipid imaging and two-photon excited fluorescence imaging of whole mount immunostaining of sciatic nerves from *SOD1*G93A versus WT. Scale bar, 20 µm. (b) Confocal microscopic imaging of whole mount immunostaining of sciatic nerves from *SOD1*G93A versus WT. Scale bar, 20 µm.

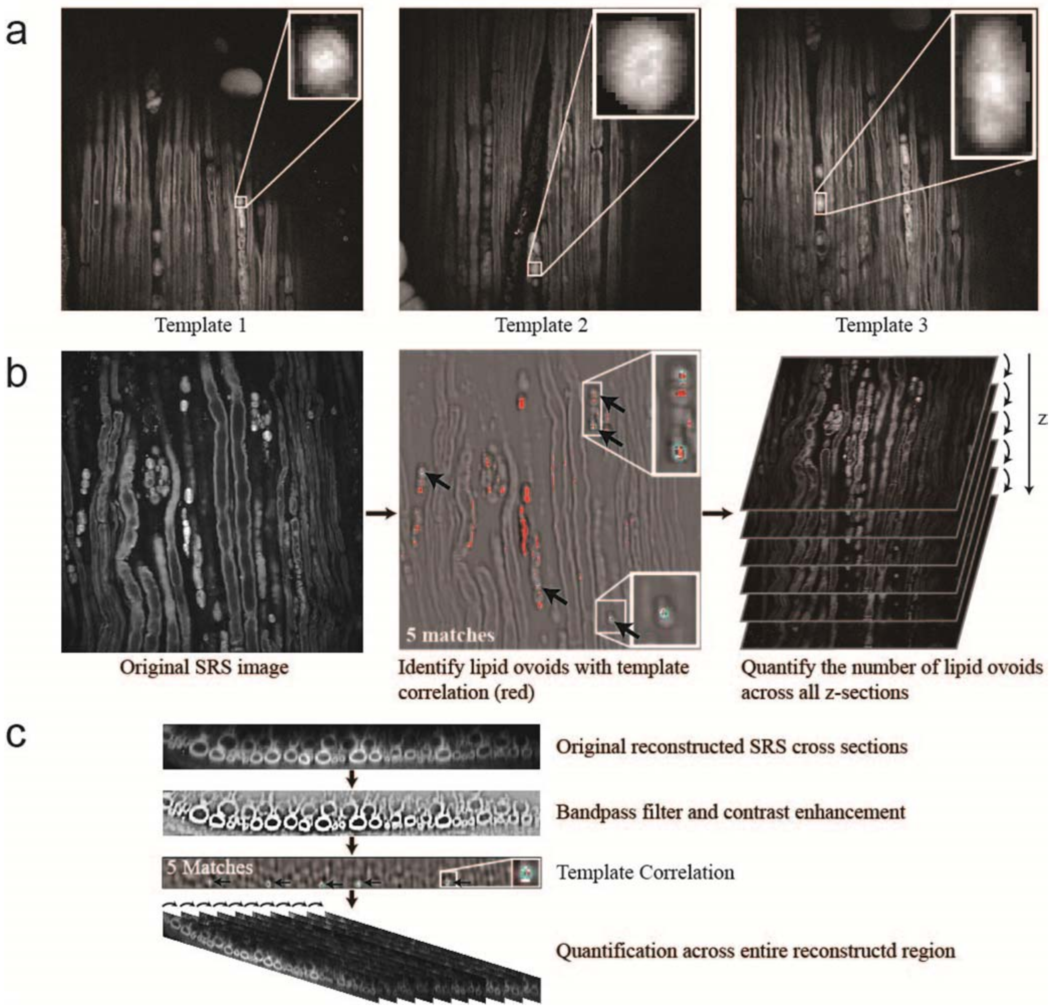

Supplementary Figure 7 Quantification algorithm of the peripheral nerve degeneration visualized by SRS imaging. (a) Template selection of lipid ovoid quantification. Three templates with representative sizes and shapes were selected. (b) Template-match algorithm for lipid ovoid quantification. The selected templates were correlated with the images. The strong correlation is shown on a threshold image. Particle counting was performed on the threshold image with roundness and area as criteria. Arrows show the identified lipid ovoids. (c) Quantification methods of nerve fiber quantification.

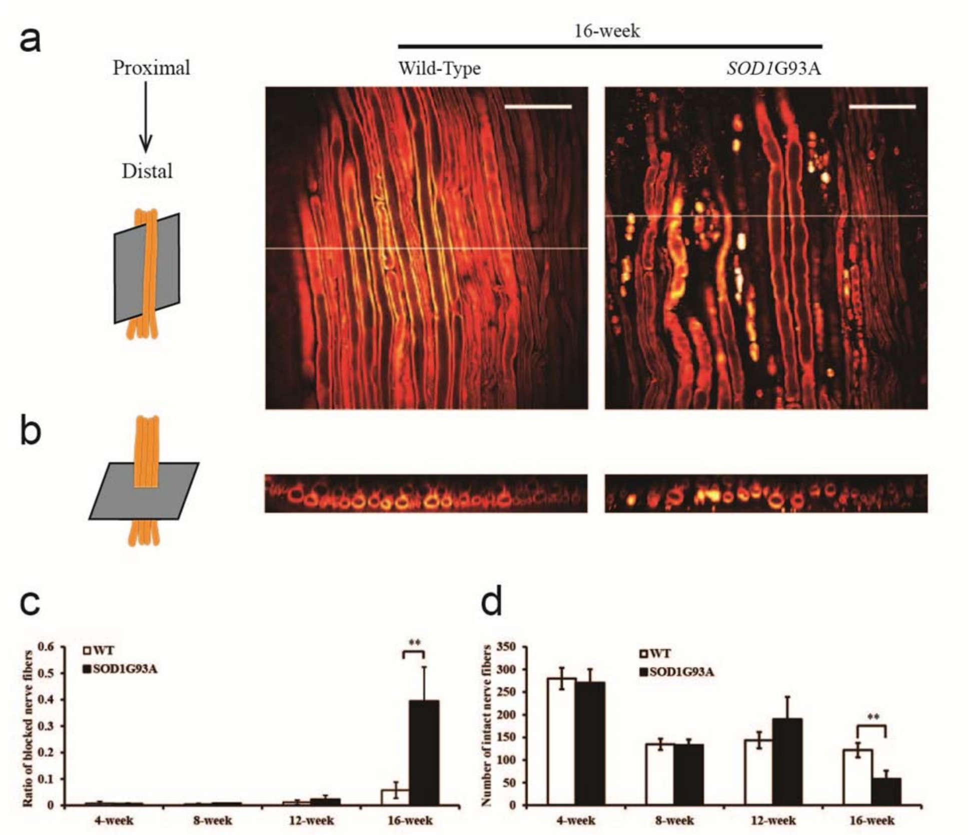

Supplementary Figure 8 3-D reconstruction of *ex vivo* SRS images and nerve fiber quantification for *SOD1*G93A mouse ALS model. (a) The longitudinal pictures of the sciatic nerve of a 16-week old *SOD1*G93A mouse. Scale bar, 50 µm. (b) The cross section image from 3-D reconstructed SRS z-stacks. Cross sections were visualized on the position indicated by the white line in (a). (c) Quantification of blockage of nerve fibers. $^{**}p<0.01$. (d) Quantification of total (blocked and intact) nerve fibers. $^{**}p<0.01$. The same number of animals as the lipid ovoid counting algorithm was quantified for each strain at the corresponding time point. No statistically significant differences were found at 4-week, 8-week or 12-week age points. For 16-week old mice, $p = 0.0030$. Data are presented as mean ± s.e.m., and error bars show s.e.m.

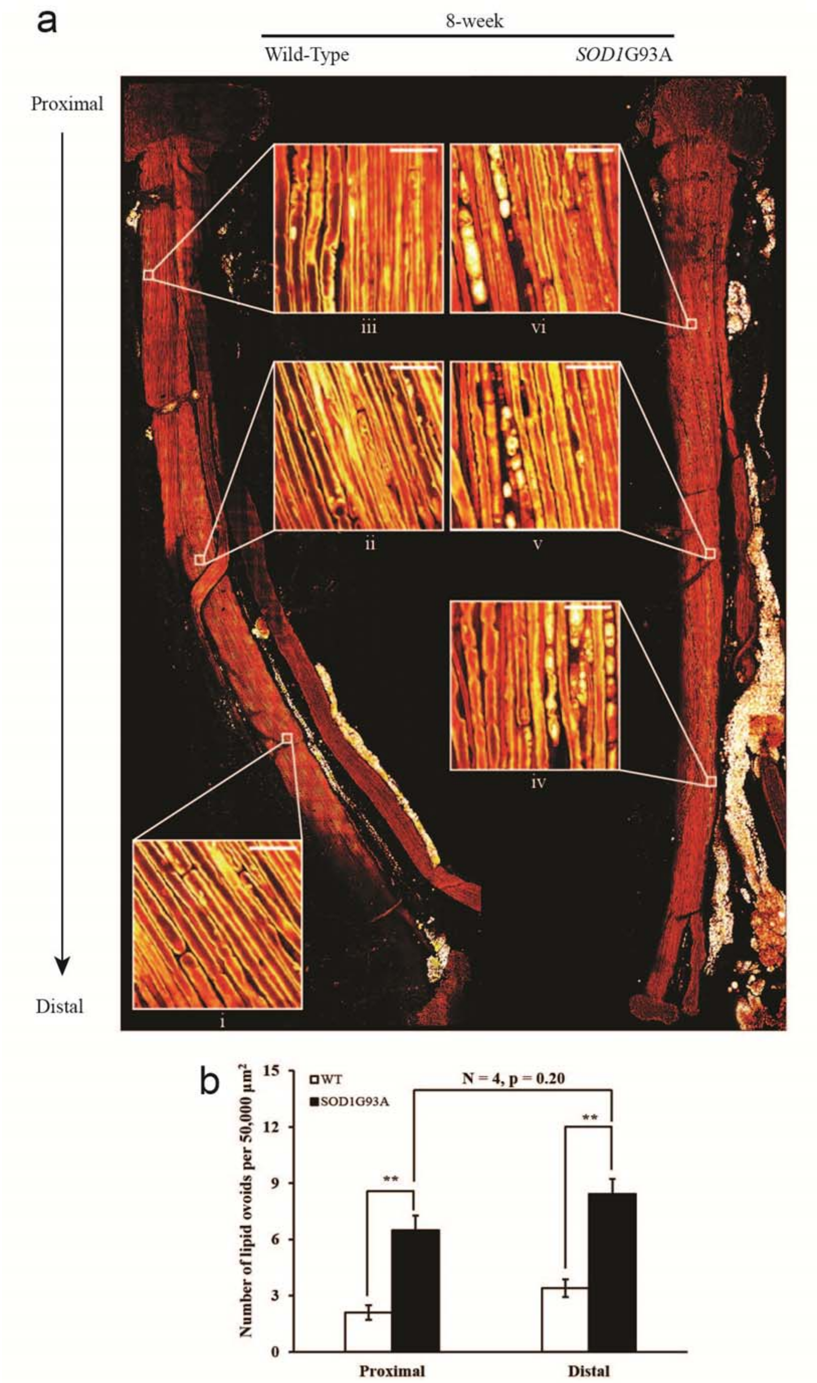

Supplementary Figure 9 Entire sciatic nerve scan of *SOD1*G93A mouse ALS model at 8 weeks postnatal. (a) Representative entire sciatic nerve scan images from 8-week *SOD1*G93A versus WT mice and zoomed in images at different locations. Scale bar, 25 µm. (b) Statistical analysis of lipid ovoid number by regions. $^{**}p<0.01$. N = 4 for each strain. $p = 0.0084$ for proximal region comparison, and $p = 0.0018$ for distal region comparison. No statistically significant difference was found between the proximal and the distal nerve in either the *SOD1*G93A mice ($p = 0.20$) or WT controls ($p = 0.17$). Data are presented as mean ± s.e.m., and error bars show s.e.m.

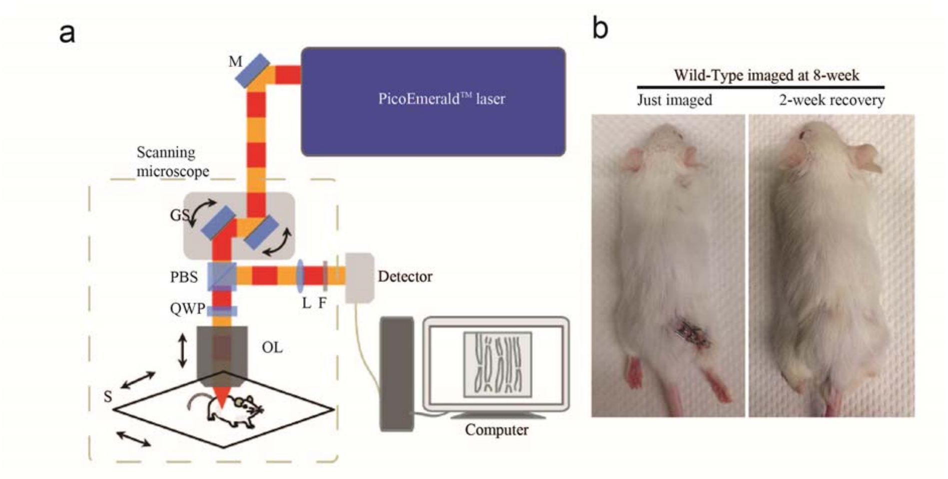

Supplementary Figure 10 *In vivo* SRS imaging device and recovery of mice after imaging. (a) Optical setup for *in vivo* SRS imaging of sciatic nerves. M: mirror, GS: galvo scanners, L: lens, F: filter, PBS: polarized beam splitter, QWP: quarter wave plate, OL: objective lens, S: motorized translation sample stage. Spatial and temporal overlapped lasers were focused on the sample by high NA water immersion objective and were scanned over the sample by an x-y galvo scanners. The z-direction movement was achieved by the z-direction movement of objective. The reflected light went through the quarter-wave plate and was reflected by polarized beam splitter and collected by the amplified silicon photodiode (Supplementary Figure 20). The electronic signal was processed and visualized on a computer monitor. (b) Incision recovery after repetitive surgery for live-mouse SRS imaging.

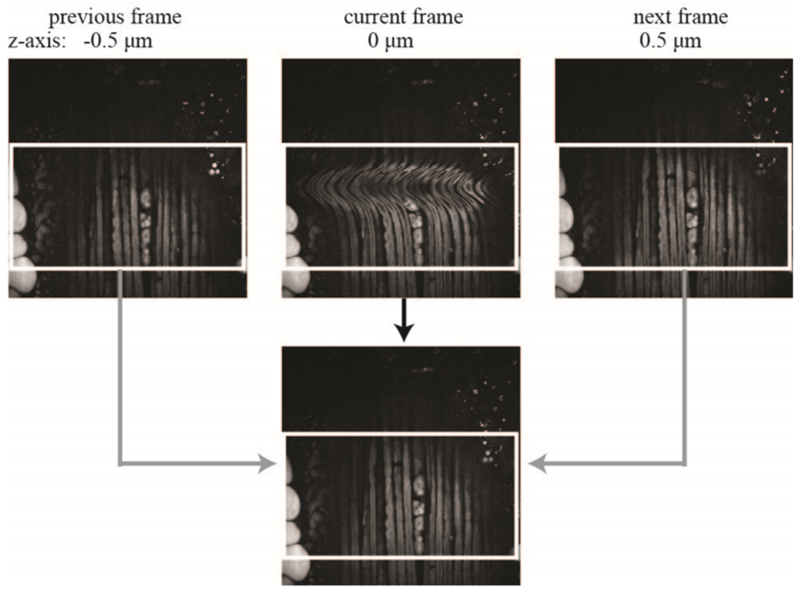

Supplementary Figure 11 Schematic of motion artifacts removing algorithm.

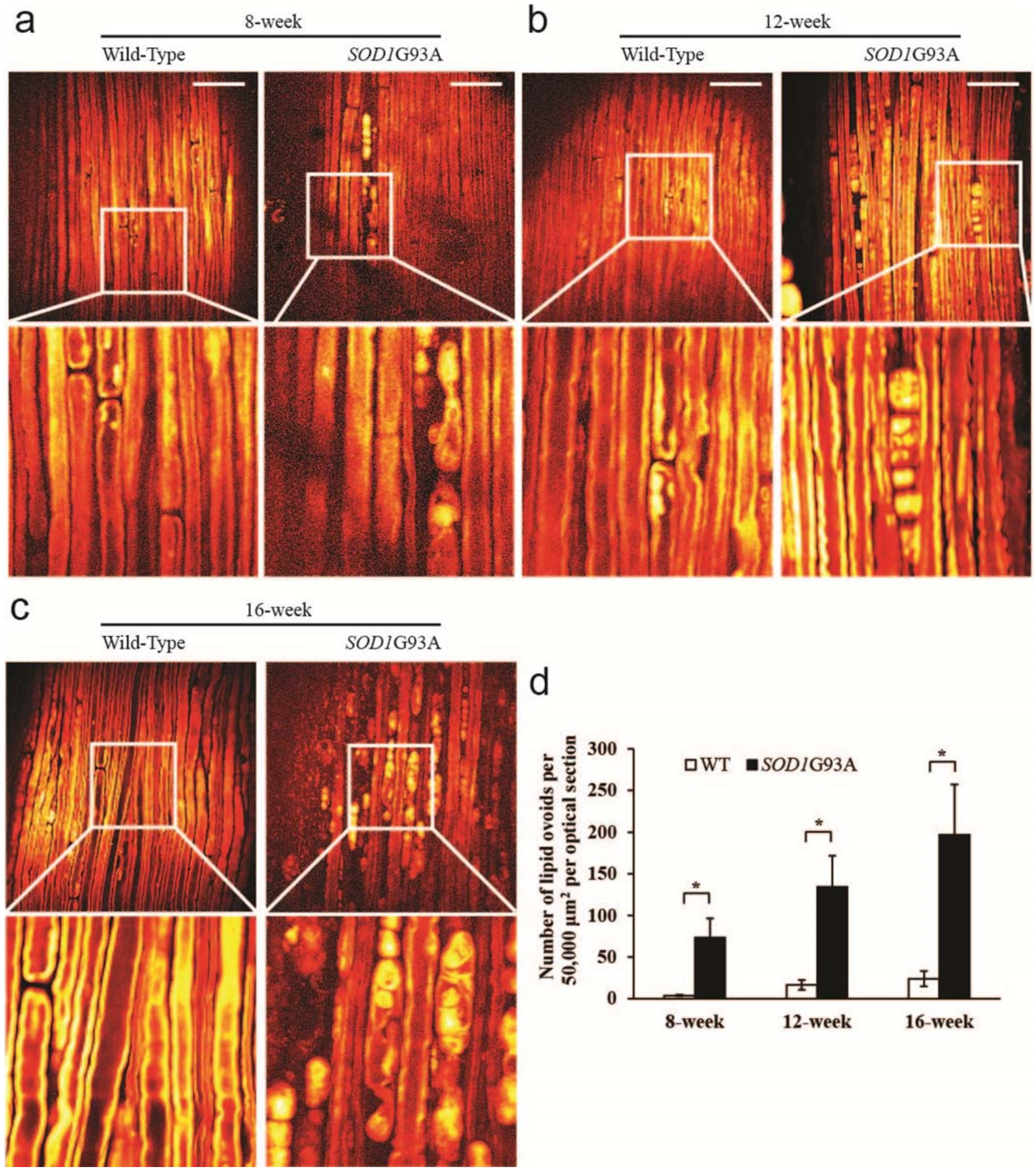

Supplementary Figure 12 *In vivo* end-point SRS imaging of anesthetized mice for *SOD1*G93A mouse ALS model. (a) *SOD1*G93A versus WT *in vivo* SRS images at 8 weeks of age. Scale bar, 50 μm. (b) *SOD1*G93A versus WT *in vivo* SRS images at 12 weeks of age. Scale bar, 50 μm. (c) *SOD1*G93A versus WT *in vivo* SRS images at 16 weeks of age. Scale bar, 50 μm. (d) Quantification of lipid ovoids for *in vivo* end-point SRS imaging. *$p<0.05$. For 8-week old mice, N = 5 for *SOD1*G93A versus N = 4 for WT, $p = 0.031$. For 12-week old mice, N = 4 for each strain, $p = 0.020$. For 16-week old mice, N = 5 for each strain, $p = 0.022$. Data are presented as mean ± s.e.m., and error bars show s.e.m.

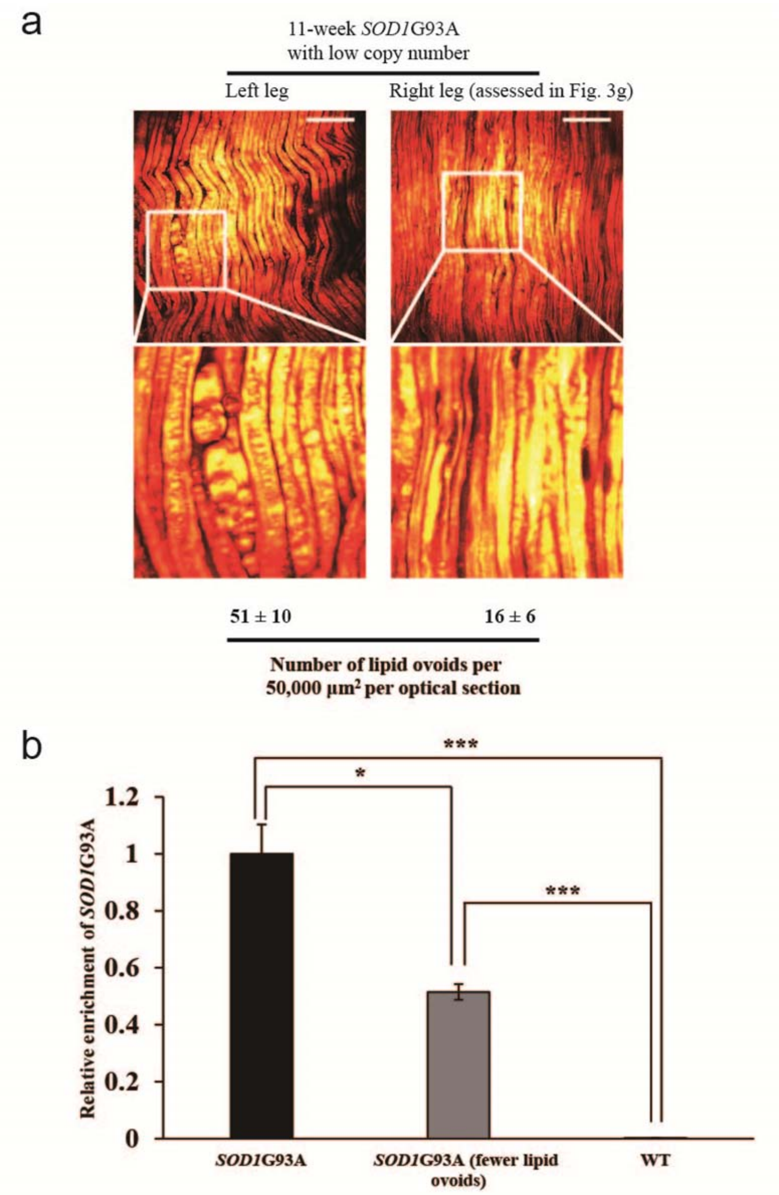

Supplementary Figure 13 Analysis of the *SOD1*G93A mouse showing fewer lipid ovoids. (a) Left versus right hind-limb SRS imaging. Scale bar, 50 μm. (b) Estimation of *SOD1*G93A transgene copy number enrichment by quantitative PCR. *$p<0.05$, ***$p<0.001$. Data are presented as mean ± s.e.m., and error bars show s.e.m.

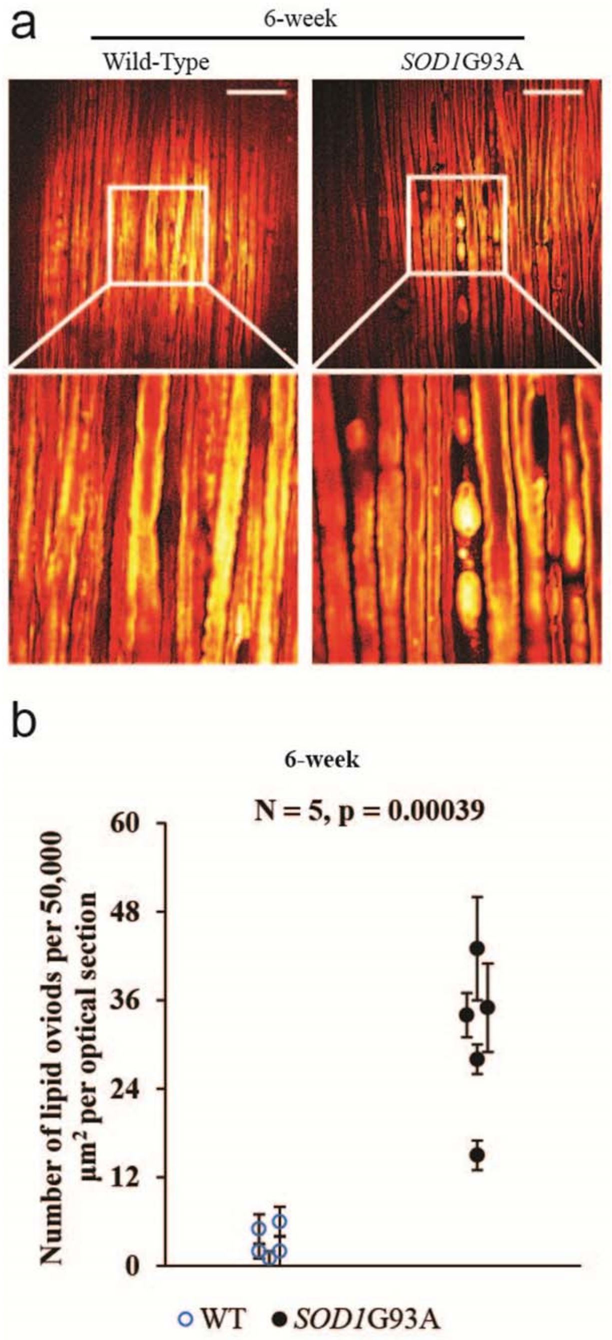

Supplementary Figure 14 6-week as the quantitatively diagnosable time point for lipid ovoid quantification methods of SRS images. (a) A representative *in vivo* WT versus *SOD1*G93A SRS images. Scale bar, 50 µm. (b) Quantification of lipid ovoids and statistical significance analysis. Data are presented as mean ± s.e.m., and error bars show s.e.m.

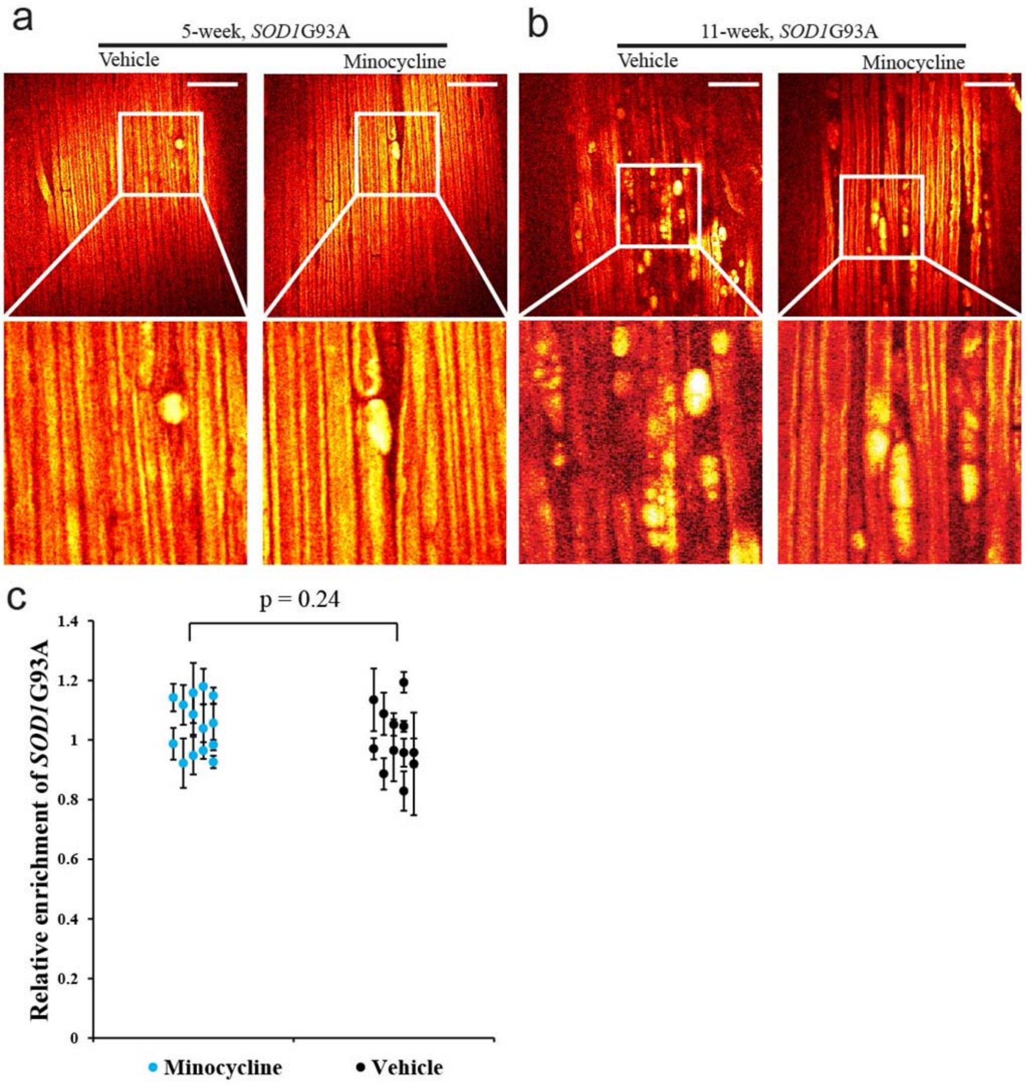

Supplementary Figure 15 Supplementary SRS imaging of *SOD1*G93A under minocycline treatment. (a) *SOD1*G93A versus WT long-term serial *in vivo* SRS images at 5 weeks postnatal. Scale bar, 50 µm. (b) *SOD1*G93A versus WT long-term serial *in vivo* SRS images at 11 weeks postnatal. Scale bar, 50 µm. (c) *SOD1*G93A copy number analysis for minocycline versus vehicle treated animals.

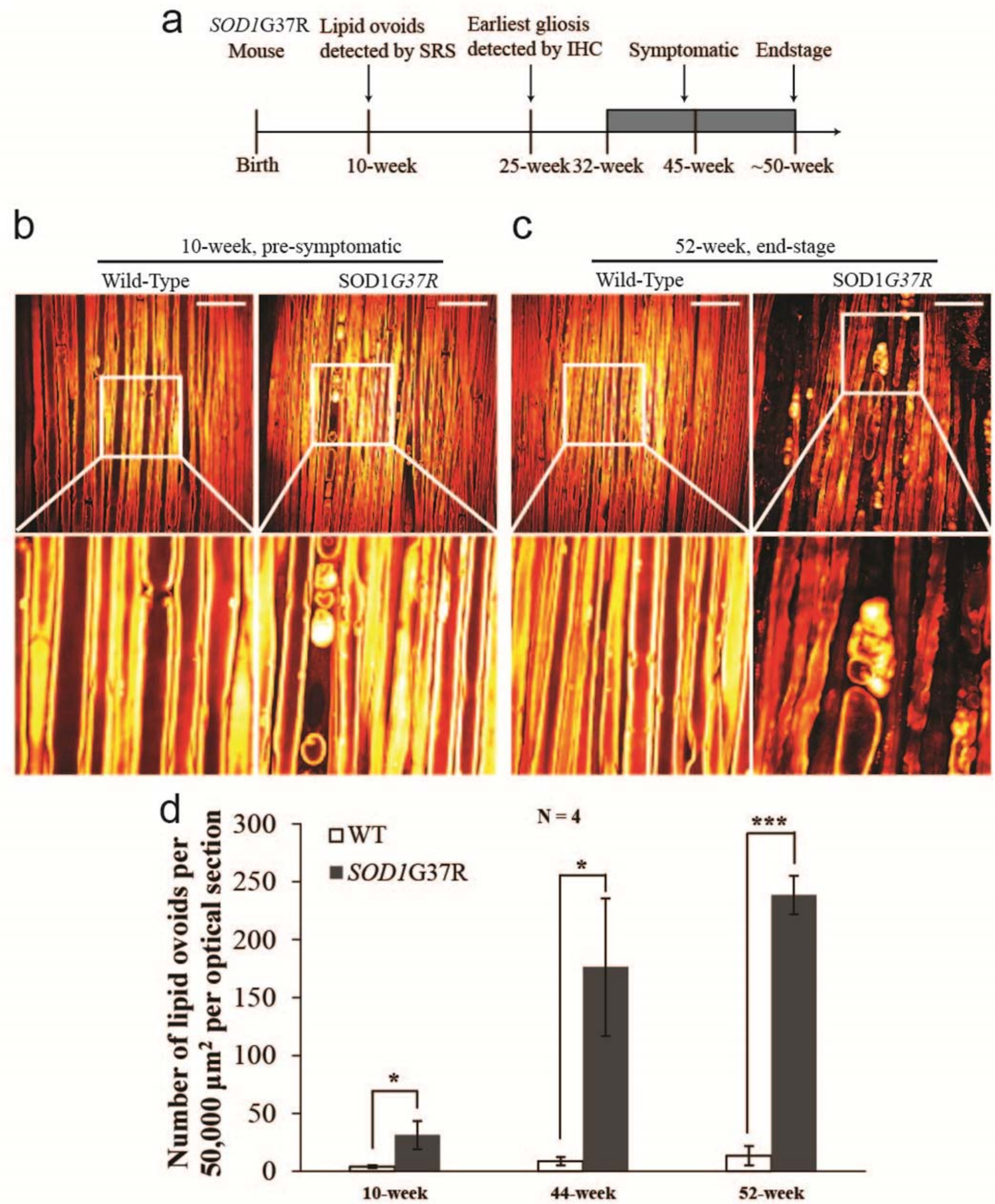

Supplementary Figure 16 SRS imaging of *SOD1*G37R mouse ALS model. (a) Disease related phenotype progression in *SOD1*G37R mouse ALS model. (b) *SOD1*G37R versus WT *in vivo* SRS images at 10 weeks of age. Scale bar, 50 μm. (c) *SOD1*G37R versus WT *in vivo* SRS images at 52 weeks of age. Scale bar, 50 μm. (d) Quantification of lipid ovoids and statistical significance analysis. $*p<0.05$, $***p<0.001$. N = 4 for each strain and each age point. For 10-week old mice, p = 0.043. For 44-week old mice, p = 0.017. For 52-week old mice, $p = 8.5\times10^{-6}$. Data are presented as mean ± s.e.m., and error bars show s.e.m.

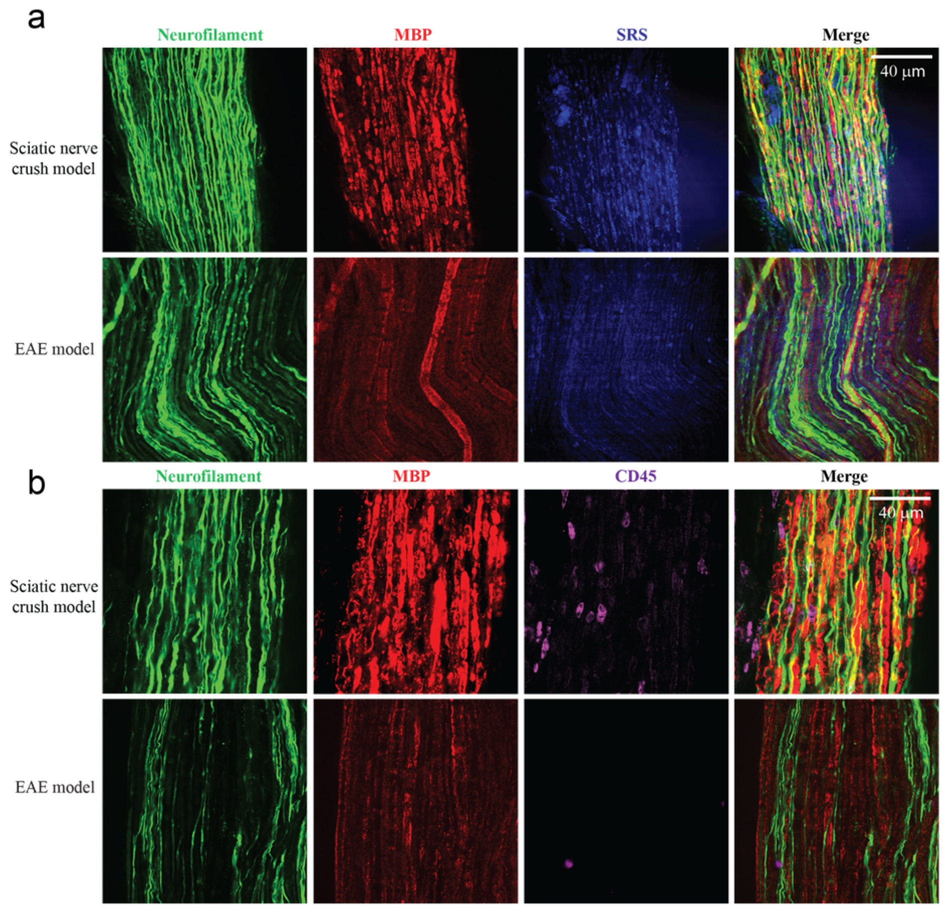

Supplementary Figure 17 Whole mount immunostaining of sciatic nerves from sciatic nerve crush model and EAE model. (a) SRS lipid imaging and two-photon excited fluorescence imaging of whole mount immunostaining of sciatic nerves from sciatic nerve crush model and EAE model. Scale bar, 40 µm. (b) Confocal microscopic imaging of whole mount immunostaining of sciatic nerves from sciatic nerve crush model and EAE model. Scale bar, 40 µm.

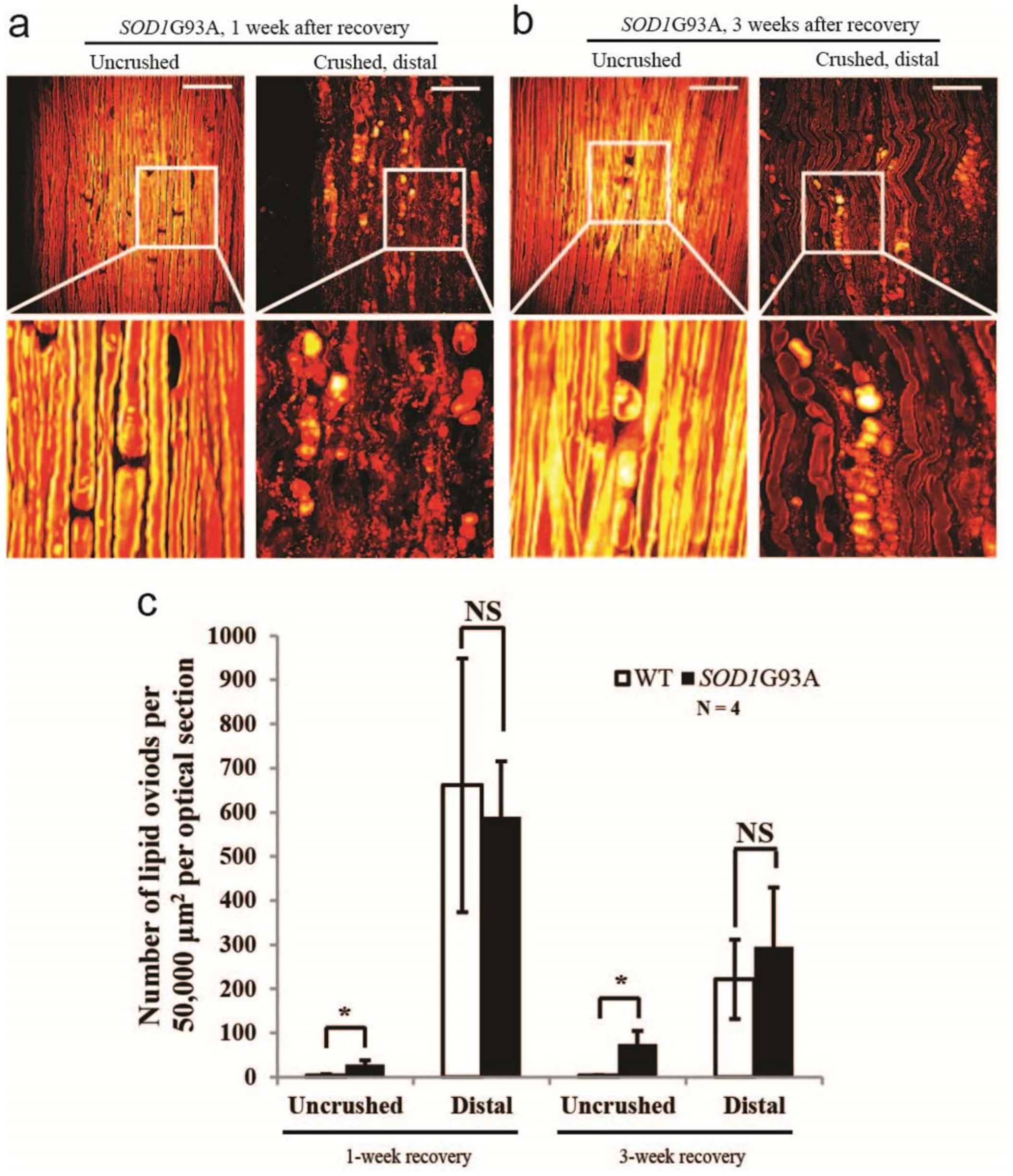

Supplementary Figure 18 SRS imaging for sciatic nerve crush model with *SOD1*G93A mouse model of ALS. (a, b) Sciatic nerve SRS images of *SOD1*G93A transgenic versus WT mice after crush and recovery. Scale bar, 50 μm. (c) Lipid ovoid quantification result of sciatic nerve crush experiment. *p<0.05. N = 4 for each strain or each age point. Data are presented as mean ± s.e.m., and error bars show s.e.m.

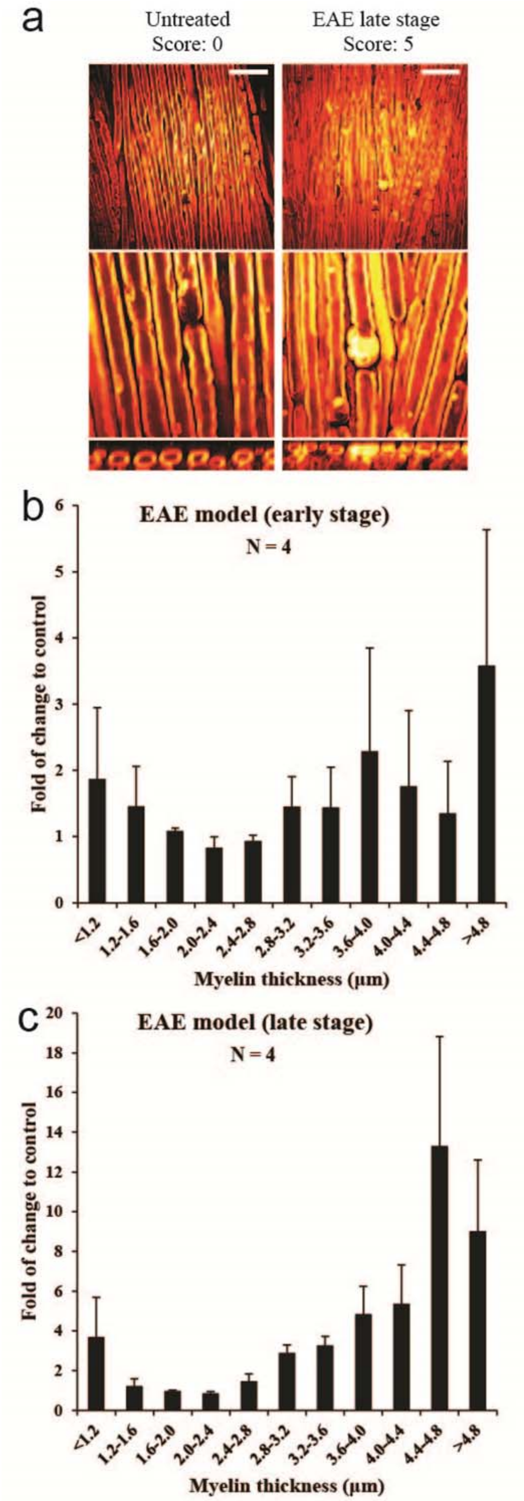

Supplementary Figure 19 Analysis of SRS imaging for EAE model. (a) 3-D reconstruction of SRS images of EAE model and identification of "onion bulb" morphology. Arrow shows a typical "onion bulb" morphology. Scale bar, 50 μm. (b,c) Myelin thickness analysis of early (b) and late (c) EAE mice. Data are presented as mean ± s.e.m., and error bars show s.e.m.

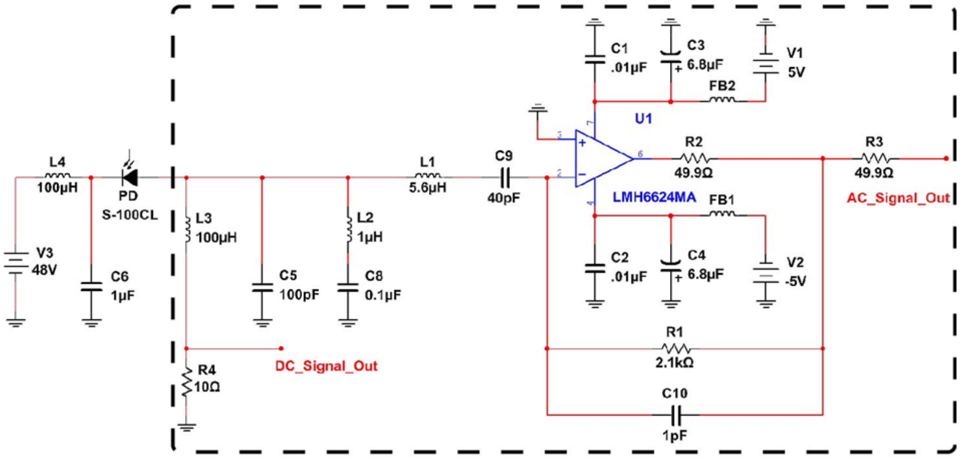

Supplementary Figure 20 The filtered transimpedance amplifier. It was designed to increase sensitivity by amplifying the signal around 10 MHz with high gain and suppressing direct current and background frequency at laser repetition rate of 80 MHz. PD: photodiode, FB: ferrite bead 75 Ω @100 MHz.

Supplementary Table 1. Comparative electromyography and in *vivo* SRS imaging results of ALS mouse model

No.	Genotype	Body Weight (g)	CMAP (mV)	Motor Unit Size (μV)	MUNE	Needle EMG	Number of Lipid Ovoids (per 50,000 μm² per optical section)	Sign of Myelin Degeneration
1	*SOD1*G93A	21.0	25.9	100.66	257.03	Abnormal (2+)	21 ± 2	Obvious
2	WT	18.2	33.1	69.88	473.67	Normal	4 ± 1	Absent
3	*SOD1*G93A	19.3	31.4	86.46	363.17	Abnormal (2+)	18 ± 2	Mild
4	WT	19.6	41.8	74.82	558.67	Normal	0 ± 0	Absent
5	*SOD1*G93A	21.9	33.3	88.32	377.04	Normal	20 ± 2	Obvious
6	*SOD1*G93A	21.0	38.5	91.68	419.94	Normal	6 ± 2	Absent
7	*SOD1*G93A	17.5	25.2	92.40	272.90	Normal	1 ± 1	Absent
8	WT	20.3	33.6	89.20	376.70	Normal	0 ± 0	Absent
9	*SOD1*G93A	19.1	45.8	90.00	500.00	Abnormal (1+)	10 ± 3	Mild
10	WT	18.4	30.0	92.00	334.00	Normal	3 ± 1	Absent

Supplementary Table 1 Comparative electromyography and *in vivo* SRS imaging results of ALS mouse model at 5 weeks of age.

Supplementary Movie 1 Mouse locomotive activity after long-term surgery.
